# An efficient method to isolate Kupffer cells eliminating endothelial cell contamination and selective bias

**DOI:** 10.1002/JLB.1TA0517-169R

**Published:** 2018-04-01

**Authors:** Ruairi W. Lynch, Catherine A. Hawley, Antonella Pellicoro, Calum C. Bain, John P. Iredale, Stephen J. Jenkins

**Affiliations:** ^1^ MRC Centre for Inflammation Research University of Edinburgh Edinburgh United Kingdom

**Keywords:** endothelial cell, flow cytometry, Kupffer cell

## Abstract

Multicolor flow cytometry and cell sorting are powerful immunologic tools for the study of hepatic mϕ, yet there is no consensus on the optimal method to prepare liver homogenates for these analyses. Using a combination of mϕ and endothelial cell reporter mice, flow cytometry, and confocal imaging, we have shown that conventional flow‐cytometric strategies for identification of Kupffer cells (KCs) leads to inclusion of a significant proportion of CD31^hi^ endothelial cells. These cells were present regardless of the method used to prepare cells for flow cytometry and represented endothelium tightly adhered to remnants of KC membrane. Antibodies to endothelial markers, such as CD31, were vital for their exclusion. This result brings into focus recently published microarray datasets that identify high expression of endothelial cell‐associated genes by KCs compared with other tissue‐resident mϕ. Our studies also revealed significant and specific loss of KCs among leukocytes with commonly used isolation methods that led to enrichment of proliferating and monocyte‐derived mϕ. Hence, we present an optimal method to generate high yields of liver myeloid cells without bias for cell type or contamination with endothelial cells.

## Abstract 2

Protocol to remove endothelial cell contamination and selection bias inherent in conventional methods for isolating and analyzing liver Kupffer cells.

**Abbreviations**
*Cdh5*: Cadherin 5
CLEC4F: C‐type lectin domain family 4 member F
KCs: Kupffer cells
LSEC: liver sinusoidal endothelial cells

## 1 INTRODUCTION

Liver Kupffer cells (KCs) are one of the largest populations of resident mϕ in the body. KCs are located in the sinusoids of the liver where they scavenge and phagocytose apoptotic cells and damaged erythrocytes,^1^ contribute to maintenance of immunologic tolerance by priming Foxp3^+^ T‐regulatory cells,^2^ and capture gut commensal bacteria that enter the circulation.^3^ Under homeostatic conditions, KCs proliferate in situ and persist with relatively little input from conventional hematopoiesis in adult mice.^4^, ^5^, ^6^ However, during liver inflammation, stress, or injury, Ly6C^hi^ monocytes are recruited to the liver and subsequently mature into monocyte‐derived hepatic mϕ. Both KCs and monocyte‐derived mϕ have been attributed prorestorative or proinflammatory roles in models of acute and chronic liver damage.^7^, ^8^, ^9^ To better understand the function of KCs and monocyte derived mϕ, it is important not only to accurately identify these cells but also to ensure a comprehensive portrait of the in vivo population is generated.

Multiparameter flow cytometry is a powerful tool for evaluating changes in number, frequency, and phenotype of diverse monocyte and mϕ populations and is the basis by which these cells are purified for subsequent functional and genomic analyses. Many protocols have been used to isolate leukocytes from the liver, but there is no consensus on which method is most valid, particularly for KCs. Furthermore, although the definition of KCs by flow cytometry is widely accepted as F4/80^hi^CD11b^lo^ cells, the reliability of this approach has not been fully investigated. For example, several microarray and RNA‐seq datasets identified *Cdh5*, a gene typically associated with endothelial cells,^10^ as differentially expressed by KCs compared with other tissue‐resident mϕ.^11^, ^12^, ^13^

Here, we demonstrate that the population of KCs conventionally defined by their F4/80^hi^CD11b^lo^ phenotype contains a significant proportion of contaminating CD45^+^CD31^hi^ endothelial cells, and that this contamination was present regardless of the method used for isolating leukocytes from the liver. Inclusion of endothelial markers rather than additional surface markers of mϕ was critical for excluding these cells from analysis. Furthermore, quantity and quality of isolated KCs varied significantly dependent on the purification method used. We therefore present a comprehensive protocol for faithfully isolating leukocytes from the liver and a modified gating strategy to effectively eliminate contaminating endothelial cells.

## 2 METHODS

### 2.1 Mice

Wild‐type (WT) C57BL/6OlaHsd CD45.2^+^, congenic C57BL/6 CD45.1^+^CD45.2^+^, C57BL/6OlaHsd *Csf1r*‐mApple^+^ mice,^14^ and *Cdh5‐*Cre*‐*ERT2 mice^15^ crossed with the mTmG (*Rosa26*Sor^tm4(ACTB‐tdTomato, EGFP)Lou^/J) line,^16^ were bred and maintained in specific pathogen‐free facilities at the University of Edinburgh, UK. Mice were sex and age matched, 6–12 weeks for WT studies and 11–12 weeks for *Cdh5* mice. Bone marrow chimeric mice were generated as previously described.^17^ Briefly, C57BL/6 CD45.1^+^CD45.2^+^ mice were anesthetized and hind legs irradiated with 950 rad while remaining tissues were protected by lead. Mice were reconstituted the next day with 2–5 × 10^6^ donor bone marrow cells from congenic CD45.2^+^ animals and rested for 8 weeks prior to analysis. All experiments were approved by the University of Edinburgh Animal Welfare and Ethical Review Body under license granted by the UK Home Office.

### 2.2 Tamoxifen administration

To induce Cre expression in *Cdh5*‐Cre‐ERT2:mTmG mice, sterile filtered tamoxifen (Sigma, Irvine, UK; 100 mg/kg) dissolved in corn oil (Sigma; 20 mg/ml) was administered i.p. for 5 consecutive days and mice left for 2 weeks.

### 2.3 CSF1‐Fc and BrdU administration

An Fc conjugate of porcine CSF1 (CSF1‐Fc) was prepared as described.^18^ Analysis of KC and endothelial cell proliferation in response to administration of CSF1‐Fc was performed on cells from a larger unpublished study aimed at assessing the effect of chronic CSF1 delivery on KCs origin in tissue‐protected bone marrow chimeric mice made as described previously.^17^ Tissue protected chimeric mice were given 1 μg/g CSF1‐Fc delivered in PBS s.c. or PBS control on day 1, 3, 17, 19, 33, and 35 before analysis on day 37. Mice were pulsed with 1 mg BrdU s.c. 2 h before necropsy.

### 2.4 Isolation of leukocytes from liver

Following perfusion of PBS through the inferior vena cava, livers were placed into RPMI, finely chopped using a razor blade and digested in 5 ml of enzyme mix (RPMI with 0.625 mg/ml collagenase D, 30 U/ml DNase [Roche, Burgess Hill, UK], 0.85 mg/ml collagenase V [Sigma], and 1 mg/ml dispase [Invitrogen, Paisley, UK] for 25 min at 37°C, in an orbital shaker with additional manual shaking every 5 min. Digests were poured through a 100 μm strainer and then prepared according to the protocols below. Unless otherwise stated, all wash buffers were kept at 4°C. At the end of all protocols, cell pellets were resuspended, passed through a 40 μm strainer, and live cells counted using a Casey TT counter (Roche).

### 2.5 300 *g* centrifugation

Cells were washed in 50 ml, then 30 ml RPMI, and centrifuged at 300 *g* for 5 min, maximum break and accelerator. RBC lysis buffer (Sigma; 2 ml) was added for 2 min, followed by 2 ml FACS buffer (PBS supplemented with 0.5% BSA and 2 mM EDTA). Cells were pelleted (300 *g*, 5 min) and the supernatant discarded.

### 2.6 33% Percoll™ gradient

Cells were washed twice in 50 ml liver wash buffer (PBS/2% FCS) by centrifugation at 443 *g* for 6 min, maximum break and accelerator. The pellet was resuspended in a room‐temperature 33% Percoll gradient (25 ml per sample) and spun at 693 *g* for 12 min, with minimum break and accelerator. The cell pellet was washed in 30 ml liver wash buffer at 300 *g* for 5 min. RBC lysis buffer (5 ml) was added for 5 min, then 30 ml liver wash buffer and cells spun at 300 *g* for 5 min.

### 2.7 50 *g* centrifugation

Cells were washed in 15 ml RPMI containing 10% FCS and centrifuged at 50 *g* for 10 min with minimum break. The supernatant was collected and spun at 340 *g* for 10 min, minimum break. The pellet was lysed for 5 min in 2 ml RBC lysis buffer on ice, topped up with RPMI + 10% FCS and spun at 340 *g* for 10 min, minimum break.

### 2.8 Collection of discarded fractions

For the 300 *g* spin and Percoll gradient methods, the supernatant or both the hepatocyte layer, and the supernatant between the hepatocyte layer and the leukocyte pellet, respectively, was collected into a fresh tube and centrifuged at 400 *g* for 5 min. The resultant pellets were counted and stained. For the 50 *g* slow‐spin method, the pellet generated following the 50 g spin was counted and stained.

### 2.9 Isolation of leukocytes from lung

Perfused lungs were collected into RPMI, homogenized using scissors and digested in 2 ml of the enzyme mix detailed above, for 45 min at 37°C. Digests were filtered through a 100 μm strainer, washed with FACS buffer and RBC lysed in 3 ml RBC lysis buffer (Sigma) for 3 min. After washing, cells were passed through a 40 μm strainer and counted.

### 2.10 Flow cytometry

2 × 10^6^ liver cells, or 20 μl of whole blood was incubated with Zombie Aqua fixable viability dye (Biolegend, London, UK) for 10 min at RT and then with 0.025 μg anti‐CD16/32 (2.4G2; Biolegend) in 10% normal mouse serum (Life Technologies, Paisley, UK). Cells were then incubated with antibodies (Supplemental Table 1). Cells were washed, spun at 300 *g* for 5 min and, where necessary, incubated with fluorescently labeled streptavidin. 7‐AAD solution (Biolegend) was added to samples 10 min before acquisition when comparing isolation protocols. DAPI was used as a viability marker for FACS. Liver cells were gated as shown, whereas alveolar and interstitial mϕ were identified as CD45^+^CD11c^+^SiglecF^+^ and CD45^+^CD11c^+^SiglecF^−^MHCII^+^CD64^+^ cells, respectively.

For BrdU and Ki67 staining, cells were fixed and permeabilized overnight in FoxP3/Transcription Factor Staining Buffer (eBioscience). Cells were washed in PermWash (eBioscience) and stained with anti‐Ki67 and anti‐BrdU antibodies.

Cells were acquired on a LSRFortessa (BD Biosciences, Wokingham, UK) or FACSAriaII (BD) at the QMRI Flow Cytometry and Cell Sorting Facility, University of Edinburgh, and data analyzed in FlowJo software (Tree Star, Ashland, Oregon). Fluorescence‐minus‐one controls were used to set gates.

### 2.11 Immunofluorescence

The median lobe from perfused liver of *Cdh5*‐Cre‐ERT2:mTmG mice was fixed in 4% methanol‐free PFA (Thermo Scientific, Paisley, UK) at 4°C for 2–3 h then washed in PBS and resuspended in 15% sucrose gradient for 1 h at room temperature followed by 30% sucrose at 4°C overnight. Tissue was then flash frozen in optimal cutting temperature (OCT) embedding matrix (Fisher Scientific, Loughborough, UK) and stored at −80°C. Seven‐micrometer sections were cut using a cryostat.

Following blocking with 20% normal goat serum, sections were stained with rat anti‐mouse F4/80 (Abcam, Cambridge, UK; CI:A3‐1) then AlexaFluor‐594 goat anti‐Rat IgG secondary (Abcam). Total number of F4/80^+^ KCs were enumerated per liver slice and GFP and F4/80 coexpression assessed. Immunofluorescent analysis of single cells was performed on FACS‐purified cells. Ten microliters of sorted cells was plated on a perfusion open and closed chamber for analysis at 20× magnification and images acquired using a Leica confocal SP5 microscope.

### 2.12 Statistical analysis

Data were analyzed in Prism 6 or 7 (GraphPad), with statistics detailed in relevant figure legends. *P < 0.05, **P < 0.01, ***P < 0.001, ****P < 0.0001.

## 3 RESULTS

### 3.1 F4/80^hi^CD11b^lo^ KCs identified by flow cytometry contain a subset of Cdh5^hi^CD31^hi^ cells

**Figure 1:**
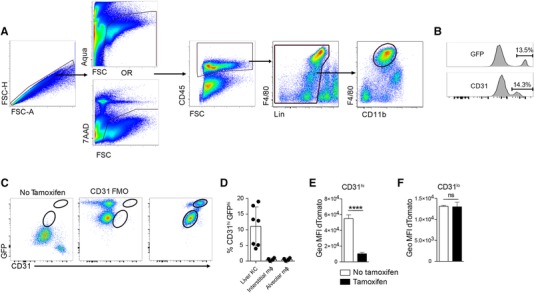
**(A) Identification of F4/80^hi^CD11b^lo^ KCs in murine liver**. Lineage = CD3, CD19, Ly6G, and Siglec F. (B) Representative GFP and CD31 expression by F4/80^hi^CD11b^lo^ liver cells from *Cdh5*‐Cre‐ERT2;mTmG mice. Proportion of GFP^+^ and CD31^hi^ cells shown (mean, *n* = 7, 3 experiments). (C) Representative GFP and CD31 expression by F4/80^hi^CD11b^lo^ liver cells from *Cdh5*‐Cre‐ERT2;mTmG mice ± tamoxifen. (D) The frequency of CD31^hi^GFP^+^ cells amongst KCs, lung interstitial and alveolar mϕ. Liver (*n* = 7, 3 experiments) and lung (*n* = 4, 2 experiments). (E and F) Geometric mean fluorescence intensity (GeoMFI) of dTomato expression by CD31^lo^GFP^−^ KCs (E) and CD31^hi^GFP^+^ cells (F) (*n* = 3/group, representative of 3 experiments)

KCs have been traditionally defined by their F4/80^hi^CD11b^lo^ phenotype, which distinguishes them from F4/80^lo^CD11b^+^ bone‐marrow‐derived myeloid cells.^19^ Global transcriptomic analyses have demonstrated that, like all tissue mϕ, KCs have a unique transcriptional signature,^11^, ^12^, ^13^ with CLEC4F and Tim4 emerging as markers that aid their discrimination from other hepatic monocytes and mϕ.^12^, ^20^ However, these same analyses also identified *Cdh5* (which encodes cadherin‐5), a gene strongly associated with endothelial cells,^10^ as part of the KC‐specific signature.^11^, ^12^, ^13^ Interrogation of the publically available ImmGen resource (http://www.immgen.org) revealed a similarly high enrichment of *Cdh5* transcripts in KCs compared with other tissue mϕ (data not shown). To establish whether KCs expressed *Cdh5*, we used a transgenic mouse strain that expresses tamoxifen‐inducible Cre recombinase under the control of the *Cdh5* promoter (*Cdh5*‐Cre‐ERT2)^15^ crossed to mTmG reporter mice.^16^ While all cells in *Cdh5*‐Cre‐ERT2:mTmG mice express tdTomato, tamoxifen administration results in Cre recombinase activity, excision of the tdTomato cassette, and an irreversible switch to EGFP expression in *Cdh5* expressing cells and their progeny. Flow cytometric analysis of whole liver isolates gated on live, CD45^+^ lineage^−^ leukocytes (Fig. 1A) revealed a clear bimodal expression of GFP in F4/80^hi^CD11b^lo^ KCs, suggesting higher expression of *Cdh5* by a fraction of these cells (Fig. 1B). Bimodal expression of the endothelial marker CD31 was also evident (Fig. 1B), and simultaneous analysis of GFP and CD31 revealed a bright double positive population (Fig. 1C) that accounted for ∼10% of the F4/80^hi^CD11b^lo^ population (Fig. 1D). In comparison, less than 0.52% of lung interstitial mϕ and 0.53% of alveolar mϕ were CD31^hi^GFP^hi^ suggesting this phenomenon was not common to all mϕ populations (Fig. 1D). Back‐gating of the CD31^hi^GFP^hi^ and CD31^lo^GFP^lo^ populations revealed they could not be discriminated based on size, granularity, and expression of other leukocyte markers such as CD45, Ly6C, and MHCII (data not shown). However, whereas tdTomato expression was significantly diminished in the CD31^hi^GFP^hi^ fraction of KCs (Fig. 1E), the CD31^lo^ GFP^lo^ cells retained identical levels to untreated control mice, indicating that Cre‐mediated excision of the tdTomato cassette had not occurred (Fig. 1F). A similar minor increase in GFP fluorescence without loss of tdTomato expression was also observed in B cells, T cells, neutrophils, and monocytes following tamoxifen treatment (data not shown). Hence, although F4/80^hi^CD11b^lo^‐defined KCs contain a subset of CD31^hi^ Cre‐expressing cells, it seems likely that the low GFP fluorescence of CD31^lo^ KC from tamoxifen‐treated mice does not represent meaningful expression of *Cdh5*‐driven Cre.

### 3.2 Endothelial cells contaminate the traditional F4/80^hi^ CD11b^lo^ KC gate

**Figure 2:**
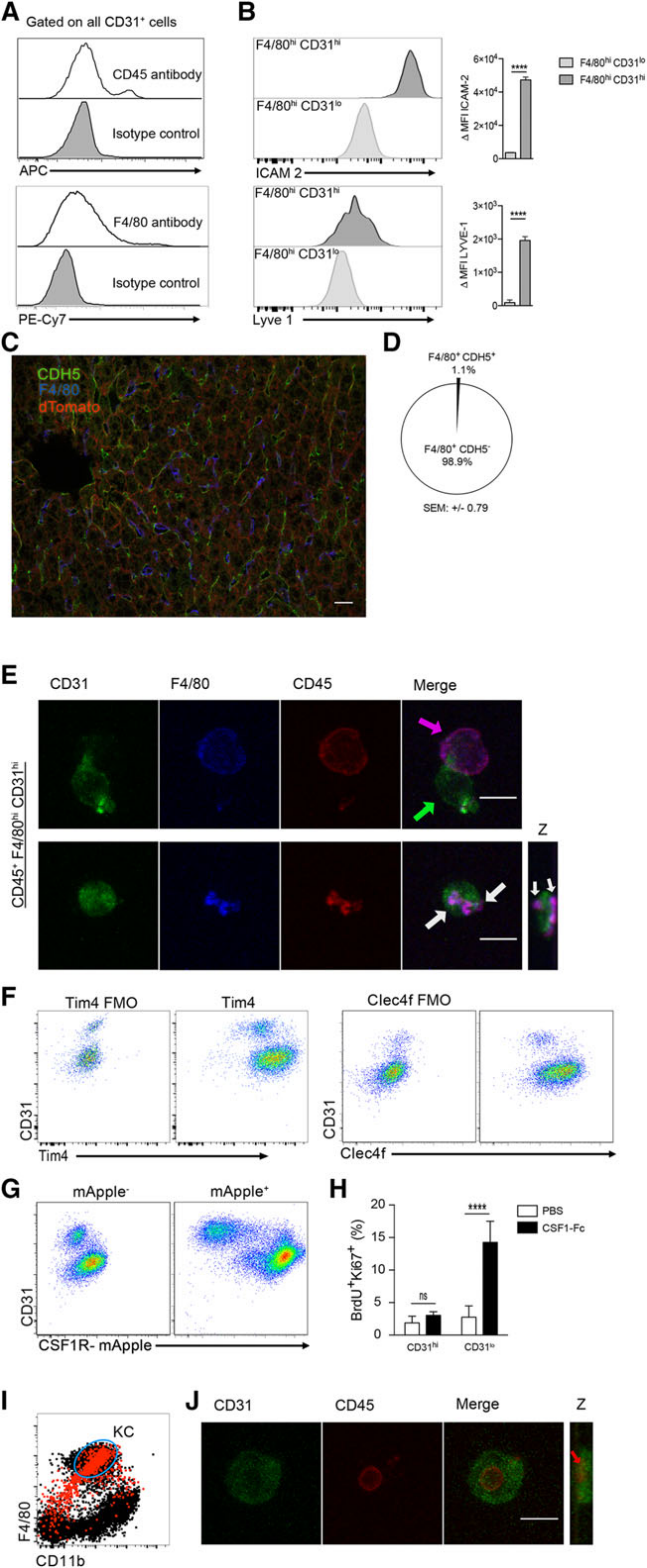
**(A) Representative expression of CD45 and F4/80 by total CD31^hi^ liver cells (*n* = 6, 2 experiments)**. (B) Representative histograms (*n* = 6, 2 experiments) and delta MFI (*n* = 3/group, representative of 3 experiments) of ICAM‐2 and LYVE‐1 expression by F4/80^hi^CD31^hi^ cells and F4/80^hi^CD31^lo^ KCs. Significance determined by *t*‐test. (C) Confocal image of liver from *Cdh5*‐Cre‐ERT2;mTmG mice (dTomato = nonrecombined cells; GFP = recombined cells) stained with F4/80 (blue). Scale bar, 20 μm. (D) Proportion of F4/80^+^ *Cdh5* ^+^ cells from (C) (10 FoV at 40× magnification of 4 livers). (E) Representative maximal projections of confocal z‐stacks of FACS‐purified F4/80^hi^CD31^hi^. CD31 (green), F4/80 (blue), CD45 (red), and merge (purple). White arrows indicate areas of punctate surface F4/80 and CD45 staining by CD31^+^ cells (*n* = 5 from 2 separate experiments). Scale bar, 10 μm. (F) Representative Tim4, Clec4f, and CD31 expression by F4/80^hi^CD11b^lo^ cells. (G) Representative mApple and CD31 expression by F4/80^hi^CD11b^lo^ cells from *Csf1r‐*mApple transgenic mice or their negative littermate controls (*n* = 9, 3 experiments). (H) Proportion of BrdU^+^Ki67^+^ cells amongst CD31^hi^ and CD31^lo^ F4/80^hi^CD11b^lo^ cells after administration of CSF1‐Fc or PBS (*n* = 4, 1 experiment). Significance determined by 1‐way ANOVA. (I) Overlay of all CD45^+^CD31^hi^ cells (red) onto CD45^+^CD31^lo^ cells. (J) Representative maximal projections of confocal z‐stacks of FACS sorted CD45^+^F4/80^−^CD31^hi^ cells, demonstrating a CD45^+^ cell (red) enveloped in an endothelial cell (green)

We further investigated the identity of the CD31^hi^GFP^+^ fraction of F4/80^hi^CD11b^lo^ cells. Analysis of all CD31^hi^ cells within the liver preparations revealed binding of CD45 and F4/80 antibodies to be specific when compared with their respective isotype controls (Fig. 2A). Unlike CD31^lo^ KCs, the CD31^hi^ F4/80^hi^CD11b^lo^ cells also expressed high levels of ICAM‐2 and Lyve‐1 (Fig. 2B), consistent with a lymphatic or liver sinusoidal endothelial phenotype. However, tissue sections from *Cdh5*‐Cre‐ERT2:mTmG mice suggested little true colocalization of F4/80 with GFP (Figs. 2C and 2D). Indeed, FACS sorting followed by confocal microscopy revealed some CD45^+^F4/80^hi^CD31^hi^ cells to be doublets of F4/80^hi^ KC and CD31^hi^ endothelium (Fig. 2E), whereas the majority of CD31^hi^ cells exhibited punctate colocalized surface staining of CD45 and F4/80 indicative of endothelial cells to which surface membrane from KC was tightly adhered (Fig. 2E). Consistent with this, staining for other KC‐specific surface markers, such as Tim4 or CLEC4f,^12^ could not differentiate the CD31^hi^ and CD31^lo^ populations despite a very marginal difference in expression intensity (Fig. 2F). In contrast, expression of a *Csf1r*‐driven mApple transgene, a cytoplasmic marker of myeloid cells,^14^ distinguished mApple^hi^CD31^lo^ KCs from mApple^−^CD31^hi^ contaminants (Fig. 2G). Furthermore, CD31^hi^F4/80^hi^CD11b^lo^ cells did not proliferate in response to the mϕ mitogen CSF1 (Fig. 2H).^18^, ^21^ Thus, it would seem most likely that CD45^+^F4/80^hi^CD31^hi^ cells identified by flow cytometry represent endothelial cells with remnants of KC membranes that are largely devoid of the intracellular components of these cells. Hence, although transgenic or intranuclear markers of KCs can aid their discrimination, surface staining for CD31 would seem the most technically simple method for excluding these cells during flow cytometry or FACS.

Of note, unbiased analysis of all CD31^hi^ cells expressing the hematopoietic marker CD45 revealed them to comprise both F4/80^hi^CD11b^lo^ cells as described above but also other hematopoietic cells (Fig. 2I). Rather than bona fide CD45^+^ endothelial cells, FACS and imaging of CD45^+^F4/80^−^CD31^hi^ cells revealed a population of endothelial cells that appeared to envelop small CD45^+^ cells (Fig. 2J). Although these could be simple doublets, they may also represent transmigrating hematopoietic cells described in human liver sinusoidal endothelial cells (LSECs) in vitro.^22^ Either way, we find no evidence that CD45^+^CD31^hi^ cells represent single cells with both hematopoietic and endothelial characteristics akin to those described in rat liver.^23^

### 3.3 Endothelial cell contamination is present irrespective of liver digestion protocol

**Figure 3:**
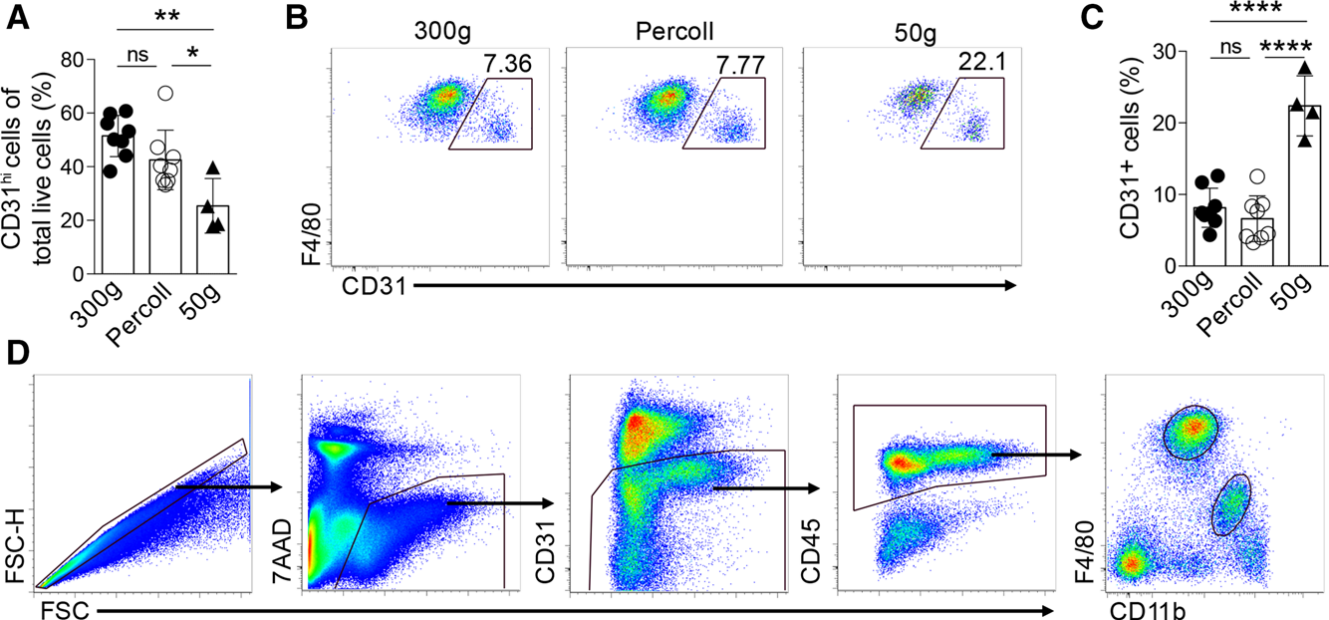
**(A) The frequency of CD31^hi^ cells of all live, single cells**. (B and C) Representative flow plots (B) and replicate data (C) of the proportion of CD31^hi^ cells amongst F4/80^hi^CD11b^lo^ cells obtained with different protocols (300 g, Percoll, 50 g) (*n* = 4–8/group, 2 experiments). (D) Improved gating strategy incorporating CD31 to identify endothelial contaminants from the F4/80^hi^CD11b^lo^ KCs population. Significance determined by 1‐way ANOVA

Multiple methods for generating single cell preparations of murine liver leukocytes have been published and while most protocols involve enzymatic digestion, the separation of leukocytes from hepatocyte debris is more inconsistent. Hence, to exclude the possibility that the observed endothelial cell contamination was an artifact specific to our protocol for isolation of leukocytes from the liver, we compared the frequency of CD31^hi^ cells in the KCs population retrieved using 3 methods representative of commonly published protocols. In brief, all methods used the same enzymatic digestion step but employed either two 300 *g* centrifugation steps (as used for Figs. 1 and 2),^17^ an initial 50 *g* prespin to first pellet and discard hepatocytes,^24^, ^25^ or a 33% Percoll gradient to remove the majority of hepatic debris.^26^, ^27^ The relative frequency of all CD31^hi^ endothelial cells recovered as a proportion of all live cells was equivalent between the 300 *g* and the Percoll gradient methods, though reduced with the 50 *g* prespin method, suggesting a proportion of endothelial cells are removed by this step (Fig 3A). However, there was a clear population of CD31^hi^ cells within the F4/80^hi^CD11b^lo^ KC gate using all 3 protocols (Figs. 3B and 3C), and most pronounced with the 50 *g* method. Notably, KC‐specific *Cdh5* expression was observed in microarray datasets generated with^11^, ^13^ or without^12^ Percoll gradient purification and low‐speed centrifugation. Hence, although it remains possible that KC express *Cdh5* at higher levels than other resident tissue mϕ, it is highly likely that contaminating endothelial cells contributed significantly to the high expression of this gene by KCs. The exclusion of CD31^hi^ cells (Fig. 3D) is therefore a critical step for the faithful identification of KCs in mouse liver irrespective of isolation protocol.

### 3.4 Isolation method can lead to selective loss of KCs

**Figure 4:**
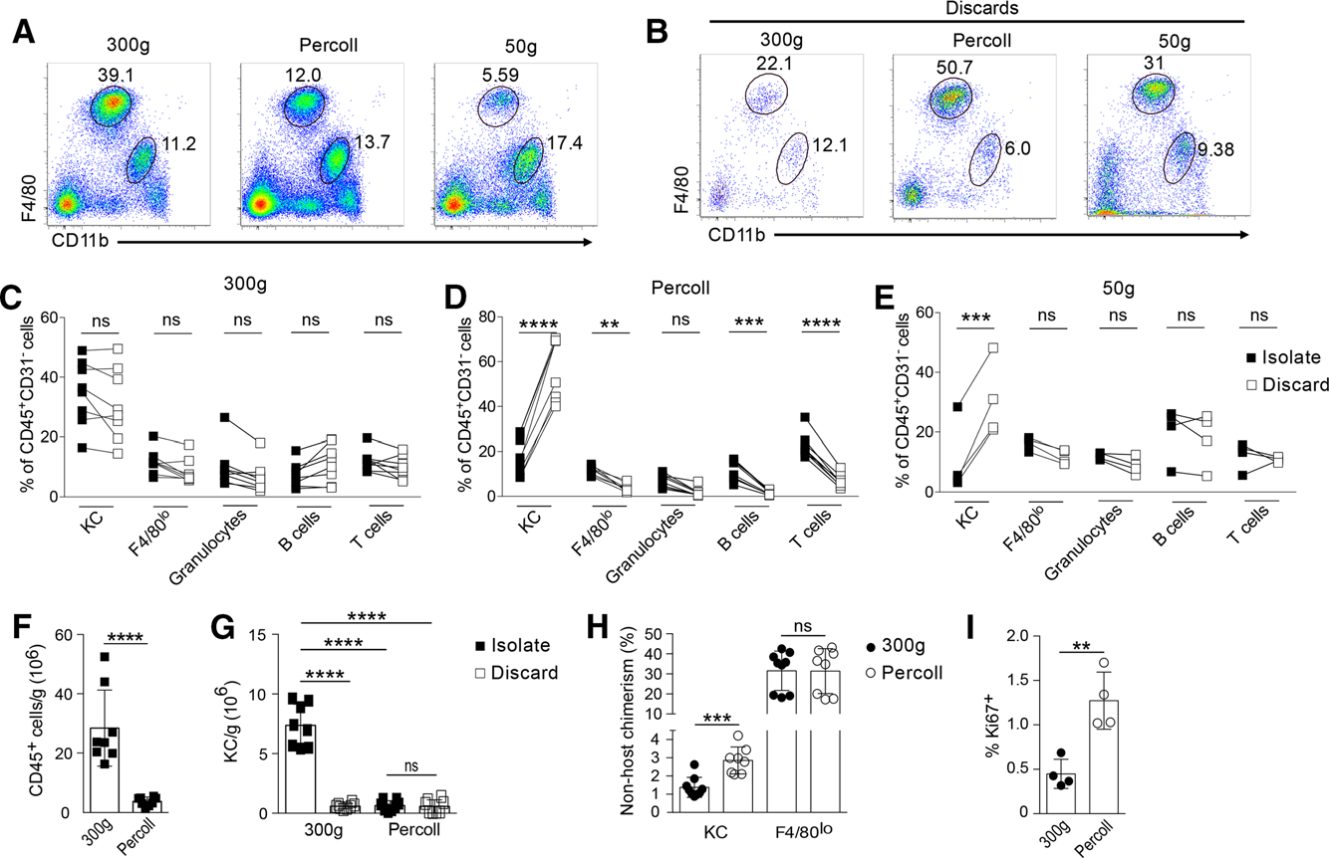
**(A and B) Representative flow plots of all isolated (A) and discarded (B) live CD45^+^CD31^lo^ cells showing proportions of F4/80^hi^CD11b^lo^ and F4/80^lo^CD11b^hi^ populations from livers prepared for flow cytometry by 1 of 3 different protocols**. (C–E) The relative frequency of F4/80^hi^CD11b^lo^ KCs, F4/80^lo^ cells granulocytes, B cells and T cells as a proportion of all CD45^+^CD31^lo^ leukocytes in the cell isolate linked to the frequency in the discard for the various protocols (*n* = 4–8/group, 2 experiments). Significance determined by multiple *t*‐test (Holm‐Sidak correction). (F and G) Number of CD45^+^ cells or CD45^+^F4/80^hi^CD31^lo^ KCs isolated by either 300 g or Percoll method (*n* = 8/group, 2 experiments). Significance determined by t‐test and 1‐way ANOVA respectively. (H) Nonhost chimerism amongst KCs and F4/80^lo^ cells from liver of WT > WT tissue‐protected BM chimeric mice isolated by 300 g or Percoll protocol (*n* = 8/group, 2 experiments). (I) Proportion of Ki67^+^ KCs isolated from liver by 300 g or Percoll protocol (*n* = 4/group, representative of 2 experiments). Significance determined by *t*‐test

After removal of CD31^hi^ endothelial cells, we observed clear differences in the relative abundance of KCs amongst live CD45^+^ cells between isolation methods, with much lower frequencies using both the Percoll and 50 *g* prespin methods, whereas the frequency of F4/80^lo^CD11b^hi^ cells was more consistent (Fig. 4A). To determine whether the difference in relative abundance in KCs between methods was due to selective loss of these cells with the Percoll gradient and 50 *g* prespin methods or enrichment with the 300 *g* method, we analyzed the hepatocyte pellet from the 50 *g* prespin, the supernatant from the first 300 *g* spin, and the hepatocyte layer from the Percoll gradient alongside the normal isolates. KCs were clearly identifiable in the discards from all protocols but at an elevated frequency in the Percoll and 50 *g* spin methods (Fig. 4B). Comparison of the frequency of KCs of total CD45^+^CD31^−^ cells within the isolate compared directly with the frequency of KCs in the discard for each sample showed that KCs were significantly enriched in the discard of both the Percoll and 50 *g* spin methods, but not in the 300 *g* spin method (Figs. 4C–4E). Thus, KCs were selectively lost using both the Percoll and the 50 *g* spin methods, whereas the ratio of all leukocytes including KCs was not altered in the discard versus the isolate with the 300 *g* spin method. The Percoll method also yielded far fewer CD45^+^ cells compared with the 300 *g* method (Fig. 4F), that together with the lower abundance of KCs, corresponded to over a 7‐fold reduction in yield of KCs using this method (Fig. 4G). It was not possible to obtain accurate cell counts from the 50 *g* method due to large amount of debris seemingly retained with this method, nor were many KCs present in the pellet extracted from the discarded Percoll (Fig. 4G), suggesting most remained within the Percoll. Importantly, the loss of a large fraction of KCs using the Percoll method also led to different interpretation of important aspects of KCs biology. Specifically, KCs turnover in CD45.1/CD45.2 congenic BM chimeras was exaggerated in Percoll preparations compared with the 300 *g* method, as was the frequency of KCs positive for the cell cycle marker, Ki67 (Figs. 4H–4I). Hence, the choice of isolation method also significantly affected readouts of KCs origin and function. Two 300 *g* centrifugation steps would appear to give the most accurate, unbiased assessment of the frequency of KCs within liver leukocytes, the greatest yield of leukocytes, and hence the most representative portrayal of the characteristics of KCs in vivo.

For simplicity, only 1 slow prespin (50 *g*) and 1 density gradient (33% Percoll) method were compared in our study. However, we acknowledge that there is considerable variation reported in number and speed of centrifugations, as well as gradient concentrations used in protocols to isolate liver leukocytes.^11^, ^13^, ^28^, ^29^ Nevertheless, our data suggest that careful optimization of these methods will be required for studies where KCs are the major cell of interest.

## 4 SUMMARY

It is imperative that populations isolated for flow cytometric, gene comparison, and functional analyses are representative of those in vivo. We have demonstrated that the established CD45^+^F4/80^hi^CD11b^lo^ gating strategy used to identify KCs in liver preparations also contains a population of endothelial cells. These cells could not be excluded using antibodies to KCs surface antigens and because endothelial cells were also found to form aggregates with other CD45^+^ cell types, we propose the simplest method to exclude them from analysis is by inclusion of antibodies to CD31 or other endothelial markers. We suggest that this strategy be adopted universally (Fig. 3D), particularly as endothelial contamination was apparent using all commonly used methods for the preparation of hepatic leukocytes for flow cytometric analysis. Cell conjugates identified by flow cytometry can reveal functionally important in vivo cell interactions^30^ and hence, endothelial cells to which KC membrane is tightly bound may well represent those LSECs intimately interacting with KCs at the point of necropsy. Since KCs remain largely static in the steady state^31^ further investigation of this subset of LSECs may identify factors controlling the turnover and function of KCs.^32^

## Supporting information

Supporting Information
